# Employment, Economic, and Sociodemographic Factors Associated with Changes in Smoking and Drinking Behaviors during the COVID-19 Pandemic in South Korea

**DOI:** 10.3390/ijerph19052802

**Published:** 2022-02-28

**Authors:** Sun Yeop Lee, Sun Kim, Woong-Han Kim, Jongho Heo

**Affiliations:** 1 JW LEE Center for Global Medicine, Seoul National University College of Medicine, Seoul 03087, Korea; sun.yp.lee@gmail.com (S.Y.L.); woonghan@snu.ac.kr (W.-H.K.); 2 Department of Global Health and Population, Harvard T.H. Chan School of Public Health, Harvard University, Boston, MA 02115, USA; sunkim1@g.harvard.edu; 3 Department of Thoracic and Cardiovascular Surgery, Seoul National University College of Medicine, Seoul 03080, Korea; 4 National Assembly Futures Institute, Seoul 07233, Korea

**Keywords:** smoking, alcohol consumption, employment status, COVID-19 pandemic, South Korea

## Abstract

The societal disruptions resulting from the coronavirus disease 2019 (COVID-19) pandemic may have caused changes in smoking and alcohol consumption. Using data from the Koreans’ Happiness Survey, a nationally representative survey in South Korea, we (1) described population-level smoking and drinking behaviors; (2) assessed changes in smoking and drinking behaviors during the COVID-19 pandemic; and (3) identified employment, economic, and sociodemographic factors associated with these changes using multinomial logistic regression. The overall amount of smoking and drinking decreased during the pandemic, but the changes were heterogeneous across subgroups. Male gender, receipt of the basic living allowance, self-employment, unemployment, and chronic disease status were associated with increased smoking, while higher household income, temporary worker status, living with someone (versus alone), and having fewer offline friends were associated with decreased smoking. Male gender, self-employment, living alone, having more offline friends, and chronic disease status were associated with increased drinking, while younger age, male gender, low and high household income (i.e., a U-shaped relationship), long-term rent with a deposit, temporary worker status, and chronic disease status were associated with decreased drinking. Our findings provide evidence on changes in smoking and drinking during the COVID-19 pandemic in South Korea and differential changes across subgroups.

## 1. Introduction

The spread of coronavirus disease 2019 (COVID-19) has not only led to a dramatic loss of human life globally, but has also broadly disrupted individuals’ lives [1,2]. Governments worldwide have implemented a variety of containment measures in response to the COVID-19 pandemic, including unprecedented lockdowns, quarantine measures, and social distancing [3]. The COVID-19 pandemic has created an environment of fear and anxiety and has led to detrimental consequences for individuals’ mental health following the mobility restrictions and minimized social interactions [4,5].

Disruptions of daily routines and social activities have also influenced individuals’ health behaviors. Specifically, recent studies have shown that the prevalence of health-harming behaviors, such as smoking or high-risk drinking, has increased during the COVID-19 pandemic in multiple countries [6,7,8,9,10,11,12,13]. From the behavioral economic standpoint, decreased constraints on substance use (e.g., in-person hours for work, school, and other obligations) and increased constraints on substance-free activities (e.g., travel, sports activities) may result in increased smoking and drinking problems [14]. However, these changes in health behaviors in the pandemic are likely to depend on aspects of the societal context, such as the severe acute respiratory syndrome coronavirus 2 (SARS-CoV-2) infection rate and national containment strategies.

In South Korea, effective COVID-19 containment strategies (referred to as ‘3T—Test, Trace, and Treat’) have slowed the SARS-CoV-2 infection rate without the need to resort to stringent lockdown measures that have incurred significant socioeconomic costs in many other countries [15]. However, because of social distancing policies such as limits on the maximum number of people at gatherings and nighttime curfews for high-risk facilities, the utilization of small businesses providing face-to-face services such as restaurants, accommodations, and fitness facilities has declined significantly, resulting in financial pressure for the self-employed and substantial overall employment losses [16]. In response, the South Korean government has sought various fiscal means, such as the COVID-19 universal transfer program, to relieve economic distress for small businesses and stimulate economic activity [17]. These societal contexts during the pandemic may have led to distinct patterns of changes in smoking and alcohol consumption in South Korea compared to other countries.

Smoking and alcohol consumption are well-known risk factors for a range of chronic diseases [18,19] and mental health disorders [20,21,22], and research has also documented strong associations between these behaviors and worse outcomes from COVID-19 [23,24]. However, household spending on alcohol and tobacco use increased by 6.2 to 10.7% in the second half of the year 2020, compared to the same time period in the previous year [25]. Moreover, changes in smoking and drinking patterns are expected to be unequally distributed [26]. Identifying subgroups of individuals who are more likely to increase or decrease their smoking or drinking would have implications for policy making in the future as well as the current pandemic. Moreover, while most studies have relied on online surveys for the surveillance of these health-harming behaviors [11,12,27,28,29,30,31,32,33], a government-led nationally representative survey is a more appropriate source for a comprehensive analysis of this context-specific phenomenon.

Therefore, the aim of this study was to (1) estimate the proportions and characteristics of the current smoking and alcohol-drinking populations in South Korea in 2020; (2) assess changes in smoking and alcohol consumption during the first year of the COVID-19 pandemic; and (3) investigate which employment, economic, and sociodemographic factors are associated with such changes.

## 2. Materials and Methods

### 2.1. Data

The Koreans’ Happiness Survey is a nationally representative cross-sectional survey that was conducted from October to December 2020 of citizens aged 15 or older [34]. For practical reasons, those living overseas or on islands, those living in dorms or special facilities, and military personnel were excluded. In total, 13,824 participants from 6857 households were selected based on a two-stage stratified cluster sampling method. A total of 650 census tracts were selected from each of 34 strata that were identified using administrative districts and strata characteristics. Ten households were randomly selected from each census tract. Professional surveyors visited the selected households, and household members aged over 15 years were provided a tablet PC to complete a self-reporting, structured questionnaire. Additional details of the survey were described in an official report [34].

### 2.2. Dependent Variables

Changes in the amount of smoking were reported by participants as “increased”, “decreased”, or “no change” compared to before the COVID-19 pandemic. The question was only answered by those who reported that they were current smokers in a preceding question. Smokers referred to people who smoked regular cigarettes, e-cigarettes, and other types of cigarettes. The “no change” category was treated as a reference category in the analyses.

Changes in alcohol consumption were measured in two ways: the frequency of alcohol consumption per week and the amount consumed per occasion. First, the participants were asked whether their frequency of drinking increased, decreased, or stayed the same compared to before the COVID-19 pandemic, or whether they did not drink alcohol. Those who identified as alcohol drinkers (i.e., those who answered “increased”, “decreased”, or “stayed the same”) also reported whether their per-occasion alcohol consumption increased, decreased, or stayed the same. Owing to the small sample size for each category of answers, a 3-point measure was created by combining information from the two questions and classifying (1) those who answered “increased” for at least one of the questions on the frequency or per-occasion consumption as “increased”; (2) those who answered “decreased” for both questions, or a combination of “decreased” and “no change” as “decreased”; and (3) those who answered “no change” for both questions as “no change”. The “no change” category was treated as a reference category in the analyses.

### 2.3. Independent Variables

Demographic variables included age, biological sex, and participants’ location of residence. Age was calculated from the birth date and treated as a continuous variable. Biological sex was self-reported as male or female. The location of residence, categorized based on the metropolitan city or province of residence, was included in the regression models to adjust for contextual factors and clustering.

Socioeconomic variables included household income, receipt of the basic living allowance, homeownership, employment type, and household type. The household income variable assessed average monthly household income before taxes in the last year. It included all household members’ income, which may include earned income, property income, and/or transfer income. It was reported in 12 categories: (1) no income; (2) less than KRW 1 million South Korean (won); (3) KRW 1–2 million; (4) KRW 2–3 million; (5) KRW 3–4 million; (6) KRW 4–5 million; (7) KRW 5–6 million; (8) KRW 6–7 million; (9) KRW 7–8 million; (10) KRW 8–9 million; (11) KRW 9–10 million; and (12) more than KRW 10 million (KRW 1 million = approximately USD 850 as of October 2021). We treated the household income variable as continuous in the main analyses. Basic living allowance receipt was an indicator of those who received the national basic living allowance at the time of the survey or in the past. The national basic living allowance, one of the social welfare programs in South Korea, is provided to those who earn less than 50% of the national median household income. As the criteria for the basic living allowance take into account the number of household members, and the allowance is only given to those without obligatory supporters, the basic living allowance variable can provide information not captured by the household income variable. Homeownership was reported in six categories: homeowner, long-term rent with a deposit, monthly rent with a deposit, monthly rent without deposit, monthly rent with a lump-sum payment, and rent without payment. In the analyses, we collapsed the last three categories into a single category of “monthly rent”, creating a three-category variable. Long-term rent with a deposit is a unique rent system in South Korea in which a large deposit is given to the landlord at the beginning of the contract and returned to the tenant at the end of the contract. Employment type was categorized into “regular employee” (the reference category), “temporary worker”, “self-employed or family business”, and “unemployed”. Regular employees included those who were on permanent contracts or had contracts lasting longer than one year. Temporary workers included those who had contracts lasting less than a year including those who worked as day laborers (i.e., workers who are hired and paid one day at a time). The “self-employed or family business” category is hereafter referred to as “self-employed” for brevity. Household type specified whether the head of household was a single father, a single mother, an underaged person, a grandparent, two parents, or the participant lived alone. None of the participants were underaged heads of household. Because of the small numbers of participants in each category, except for the “two parents” and “living alone” categories, we binarized the variable into “living alone” or “living with someone” to focus on the relationship between living alone and changes in smoking and drinking behaviors. Living alone is a variable of interest in the context of the pandemic considering the government-imposed containment measures such as lockdowns and self-quarantines.

Other independent variables included the number of offline friends and chronic disease status. Offline friends were defined as friends with whom the participant often spent time in person. Chronic disease status was reported in terms of the time since its onset: “none”, “less than 3 months”, “3–6 months”, and “more than 6 months”. The “less than 3 months” and “3–6 months” categories were combined to create a three-category variable because (1) the number of participants in the “3–6 months” category was small, and (2) the COVID-19 pandemic hit South Korea approximately 6 months before the survey, meaning that the use of a 6-month cut-off facilitated a clearer interpretation; the newly derived “less than 6 months” category includes those who developed chronic disease after the rise of COIVD-19. 

### 2.4. Statistical Analyses

Descriptive characteristics were presented separately for smokers and drinkers, as the analyses of changes in smoking or drinking only included those who smoked or drank, respectively, either before or after the pandemic. In other words, those who neither smoked nor drank before and after the pandemic were excluded from the analytic sample.

A multinomial logistic regression model was fit for the associations of each of the two dependent variables (i.e., smoking change and drinking change) with a range of independent variables. Age and household income were modeled as restricted cubic spline functions with 3 degrees of freedom (i.e., 2 knots at tertiles) to assess the possibility of nonlinear relationships with the dependent variables.

Additional analyses were conducted to check the sensitivity of the main model specifications and robustness of the main analyses. First, since the household income variable was collected as categorical instead of continuous in the survey, the 12-category variable was modeled to check for robustness. The highest income category (more than KRW 10 million) was treated as a reference category. Second, the main analyses were repeated with a sample excluding those aged 19 or younger. Under South Korean laws, teenagers can start smoking and drinking from 1 January of the year they turn 19. It is illegal for those younger than 19 to smoke and drink, so their behaviors could have been momentary rather than regular behaviors. Furthermore, the responses of those who just turned 19 may not be reflective of changes due to the pandemic since the amount of smoking and alcohol consumption would naturally rise when this group was initially permitted to smoke and drink, especially since the pandemic started at the beginning of 2020. Third, the statistical interaction between household income and employment status was modeled as a post hoc exploratory analysis.

No missing data adjustment was necessary, as there were no missing data in the analytic sample and included variables. Accounting for the complex multi-stage sampling of the participants, descriptive statistics and regression analyses were weighted by the survey weights provided in the official data set to correctly estimate population parameters for the nationally representative sample. All analyses were performed in R 4.1.1 (MathSoft, Cambridge, MA, USA) [35].

## 3. Results

Descriptive statistics were separately presented for the entire sample and each analytic sample for the two dependent variables (Table 1). Among the nationally representative sample of South Korea, 2285 individuals (16.5%) reported that they were current smokers, and 7912 individuals (57.2%) reported that they were current alcohol drinkers. Although COVID-19 infection status may have an impact on changes in smoking or drinking behaviors, only 15 participants in the entire sample (0.1%), no participants in the smoker sample (0.0%), and 9 participants in the drinker sample (0.1%) reported having tested positive for COVID-19. Because of these very small numbers, this variable was not considered in the regression analyses. Other descriptive statistics for the entire sample were described in more detail in an official report [34].

Among smokers, the mean age was 45.85 years (standard deviation (SD) = 14.93 years; range = 16–86 years), and the vast majority were male (92.5%) (Table 1). About half of the smoker sample were regular employees (57.7%), roughly a quarter were self-employed (20.4%), and 14.2% were unemployed. Approximately 1 in 10 lived alone (9.1%), and the median number of offline friends was 5 (interquartile range (IQR): 3–7). In the smoker sample, 4.4% of participants had a chronic disease for less than 6 months and 9.7% had a chronic disease for more than 6 months. The majority of smokers stated that they had not changed their amount of smoking since the pandemic (74.1%), while 6.9% reported smoking more and 19.0% reported smoking less. Among drinkers, the mean age was 44.94 years (SD = 15.47 years, range = 15–93 years), and 64.0% were male. About half of the drinker sample were regular employees (52.0%), roughly a quarter were unemployed (25.5%), and 15.6% were self-employed. A small proportion of the drinkers lived alone (6.9%), and the median number of offline friends was 5 (IQR: 3–7). In the drinker sample, 3.4% had a chronic disease for less than 6 months and 7.7% had a chronic disease for more than 6 months. Since the pandemic, the majority reported not having changed their amount of drinking (67.9%), while 4.0% reported drinking more and 28.1% reported drinking less.

**Figure 1:**
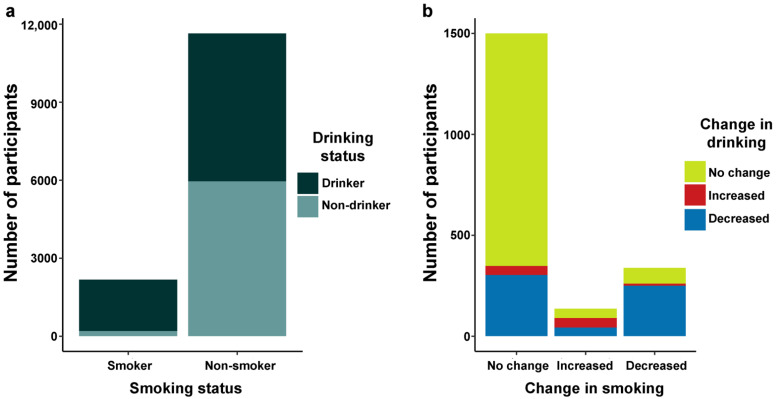
Bivariate relationships between (**a**) smoking status vs. drinking status and (**b**) change in smoking vs. change in drinking.

There was a moderate relationship between smoking and drinking, as 90.7% of smokers were also drinkers, but only 48.8% of non-smokers were drinkers (chi square (degree of freedom, df = 1) = 1300, *p* < 0.001; Cramer’s V = 0.31) (Figure 1). Changes in alcohol consumption and changes in smoking were also moderately associated (chi square (df = 4) = 647, *p* < 0.001; Cramer’s V = 0.12). Among participants who reported both smoking and drinking, the distribution of drinkers who increased their alcohol consumption by smoking category was as follows: 45.6% increased their smoking amount, 10.7% decreased their smoking amount, and 43.7% kept their smoking amount constant. Among those who drank less, 41.9% smoked less, 7.2% smoked more, and 50.8% kept their smoking constant. Finally, those who kept their alcohol consumption constant predominantly also kept their smoking amount constant (90.2%), while 3.7% smoked more and 6.1% smoked less.

Among the smoker sample, several factors were associated with smoking more or smoking less since the start of the pandemic (Table 2). Being male (odds ratio, OR = 3.20, 95% confidence interval, 95% CI [1.24, 8.26]) and receiving the basic living allowance (OR = 4.38, 95% CI [1.36, 14.05]) were associated with much higher odds of increased smoking. Compared to regular employees, those who were self-employed (OR = 2.69, 95% CI [1.73, 4.19]) or unemployed (OR = 3.26, 95% CI [1.65, 6.44]) also had a much higher likelihood of increased smoking. Furthermore, compared to those who did not have a chronic disease, a higher likelihood of increased smoking was associated with having a chronic disease for less than 6 months (OR = 2.17, 95% CI [1.11, 4.23]) or for more than 6 months (OR = 2.32, 95% CI [1.37, 3.95]).

**Figure 2:**
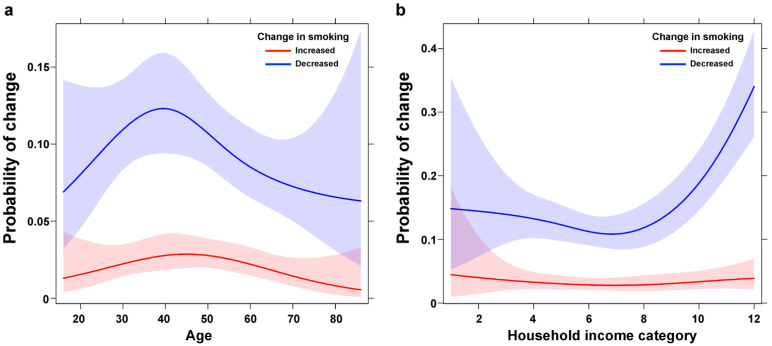
Probability of changes in smoking by (**a**) age and (**b**) household income in restricted cubic spline function. The probability of decreasing the smoking amount during the COVID-19 pandemic is higher for those with higher household income.

In contrast, temporary workers were more likely to decrease their amount of smoking (OR = 1.80, 95% CI [1.11, 2.90]) than regular employees. Living with someone instead of living alone (OR = 0.56, 95% CI [0.32, 0.98]) and having fewer offline friends (OR = 0.95, 95% CI [0.91, 0.99]) were associated with decreased smoking. Household income had a statistically significant relationship with the overall change in smoking and the visualization of the relationship indicates that higher household income was associated with higher odds of smoking less (Figure 2).

Among the drinker sample, being male (OR = 1.87, 95% CI [1.41, 2.48]), living alone (OR = 2.16, 95% CI [1.40, 3.33]), and having more offline friends (OR = 1.04, 95% CI [1.01, 1.08]) were associated with higher odds of increased drinking. Compared to regular employees, those who were self-employed had about twice the odds of drinking more (OR = 2.20, 95% CI [1.60, 3.03]). Those who had a chronic disease for less than 6 months (OR = 2.16, 95% CI [1.29, 3.61]) or more than 6 months (OR = 2.26, 95% CI [1.51, 3.36]) were also more likely to increase their amount of alcohol consumption.

**Figure 3:**
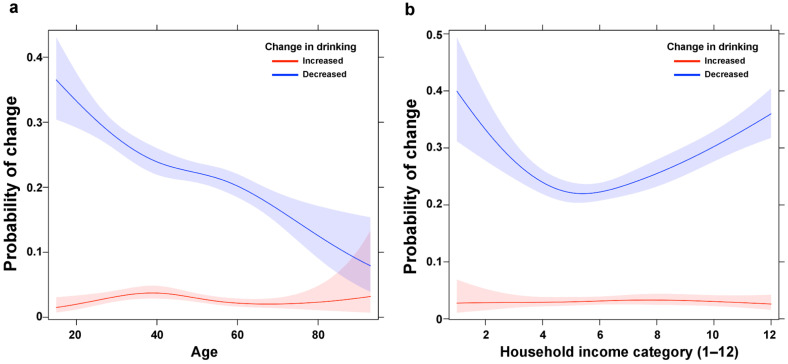
Probability of changes in drinking by (**a**) age and (**b**) household income in restricted cubic spline function. The probability of decreasing the drinking amount during the COVID-19 pandemic was higher for those with younger age, and low and high household income (i.e., a U-shape relationship).

Many factors were associated with a decrease in alcohol consumption. Being male (OR = 1.57, 95% CI [1.39 1.77]) and a higher household income (OR = 1.06, 95% CI [1.03, 1.09]) were associated with drinking less since the start of the pandemic. Compared to those without a chronic disease, those who had a chronic disease for less than 6 months were more likely to reduce their amount of drinking (OR = 1.63, 95% CI [1.23, 2.16]). Temporary workers were more likely to decrease their alcohol consumption than regular employees (OR = 1.28, 95% CI [1.03, 1.60]). Homeowners were more likely to decrease their alcohol consumption than those who had long-term rent with a deposit (OR = 0.77, 95% CI [0.66, 0.88]). A U-shaped relationship with household income was found, indicating that both higher and lower household income, compared to middle income, were associated with a higher probability of drinking less (Figure 3). Younger age was also associated with a higher probability of drinking less.

When the household income variable was treated as categorical, there was no major change in the findings (Table S1). Specifically, the highest income category (KRW 10 million or more) had higher odds of smoking less than all the other income categories. For the drinker sample, the U-shaped relationship persisted, as those with higher (i.e., KRW 9 million or more) or lower household income (i.e., KRW 1 million or less) had higher odds of drinking less than those with middle income.

When we restricted the analytic sample to those aged 20 or above, 11 participants from the smoker sample and 112 participants from the drinker sample were excluded. However, the age-restricted subgroup analysis yielded findings that were consistent with the main analyses (Table S2).

**Figure 4:**
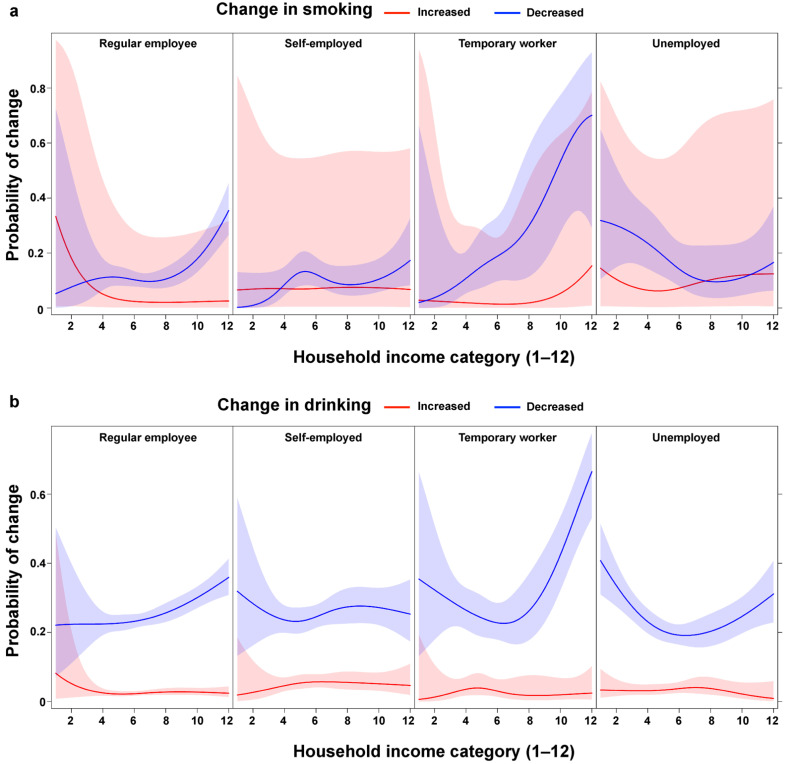
Probability of changes in (**a**) smoking and (**b**) drinking by household income within employment statuses. The interaction terms are statistically significant for both smoking changes *χ*^2^ ((df = 18) = 51.5, *p* < 0.001) and drinking changes (*χ*^2^ (df = 18) = 33.9, *p* = 0.013).

The relationships between employment status and smoking and drinking were further investigated by testing the statistical interaction with household income. The interaction was statistically significant for smoking change (Figure 4). The trend for increasing probability of smoking less with increasing income was consistent across regular employees and temporary workers, but for the self-employed and the unemployed, the probability of smoking less did not vary by income. Instead, for the self-employed and the unemployed, the probability of smoking more was as high as the probability of smoking less. In addition, the probability of reducing smoking was highest for temporary workers with higher household income. The interaction between employment status and household income for drinking change was also statistically significant. The U-shaped relationship between household income and reduced drinking was observed for temporary workers and the unemployed, but not for regular employees and the self-employed. Again, the probability of reduced drinking was highest for temporary workers with higher household income.

## 4. Discussion

As the COVID-19 pandemic has brought dramatic changes to individuals’ daily lives, subsequent changes in health-harming behaviors should be monitored on the national level for public health purposes. In addition, understanding heterogeneity in these behavioral changes is important for policy making. In a nationally representative sample of South Korea in 2020, we investigated changes in smoking and alcohol consumption since the start of the COVID-19 pandemic and identified factors associated with these changes. We found that, on average, the amount of smoking and drinking decreased during the pandemic, but the changes were heterogeneous across subgroups. Especially under the social distancing measures that directly reduced opportunities for smoking and drinking in social settings, the differential increases in certain subgroups are noteworthy. Male gender, receipt of the basic living allowance, self-employment, unemployment, and chronic disease status were associated with increased smoking, while higher household income, temporary worker status, living with a family member, and having fewer offline friends were associated with decreased in smoking. Regarding changes in alcohol consumption, male gender, self-employment, living alone, having more offline friends, and having a chronic disease were associated with a higher likelihood of increased drinking, compared to no change, while younger age, male gender, high or low household income (i.e., a U-shaped relationship), long-term rent with a deposit, temporary worker status, and having a chronic disease were associated with a higher likelihood of decreased drinking.

Employment conditions had strong and consistent associations with individuals’ changes in smoking or drinking behaviors during the pandemic even after controlling for individuals’ economic conditions. The increase in smoking and drinking for those in insecure employment situations may reflect strategies to cope with the increase in mental distress due to financial and work-related pressure [36,37]. Social distancing measures in South Korea, such as the limit on the maximum number of people at gatherings and nighttime curfews, brought financial hardships, especially for the self-employed [38]. Moreover, the unemployment rate in small establishments and for low-skill workers substantially increased during the pandemic [16]. However, for most of 2020 in South Korea, the only employment safety net (the Unemployment Insurance Scheme) did not offer any financial protection for the self-employed, small business owners, or precarious workers [39]. In December 2020 (i.e., after the current survey), to address the welfare blind spots and mitigate these financial impacts, the National Employment Insurance and the National Support for Employment were put into effect. Whether these new welfare programs have successfully reduced mental distress and related health-harming behaviors should be investigated.

Economic conditions had more nuanced relationships with changes in smoking and drinking behaviors. The higher likelihood of reduced smoking and drinking for those with high household income may be explained by various work-related factors that were uncontrolled in the current analyses, including essential worker status and flexibility in terms of remote work and work hours. Those with low household income also reduced their drinking (i.e., there was a U-shaped relationship between income and drinking reduction). Holding employment conditions constant, reductions in overall spending, including spending on alcohol and smoking, are expected for low-income households during financially strained times. However, those who received the national basic living allowance were more likely to increase their smoking, reflecting a different behavioral pattern than those with only low household income. Further investigations of behavioral changes in this subgroup are warranted with different data and a larger sample size.

Living alone and having more offline friends tended to promote smoking and drinking behaviors during the pandemic. Living alone rather than together with family members may provide more opportunities for smoking and drinking alone and exacerbate loneliness and boredom, which are already heightened with the pandemic containment measures [40,41]. However, those with more offline friends would have more opportunities to smoke or drink at gatherings, possibly in non-adherence to social distancing and nighttime curfew policies [42].

The disproportionate increase in smoking and drinking in chronic disease patients illuminates the negative impacts of the pandemic on this subgroup. The extended period of social distancing restrictions and pandemic-related shortage of healthcare resources disrupted the delivery of chronic disease care and compromised the quality of disease management [43,44]. However, those diagnosed with a chronic disease within 6 months prior to the survey were also more likely to decrease drinking than to have no change compared to those without chronic disease, suggesting that a recent diagnosis of chronic disease may also lead to beneficial changes in health behaviors. These variables (i.e., having a chronic disease for 6 months or less, male gender, and household income) with simultaneous associations with both increased and decreased risk behaviors, compared to a lack of a change, reflect heterogeneity in responses even within these subgroups, calling for intersectional analyses in future studies.

Our study has some limitations. First, it used cross-sectional data, and the baseline amount of smoking and drinking was not available. A comparison of behavioral data obtained before and after the pandemic would be better for estimating the impact of the pandemic, but data of that type are not available for a nationally representative sample. Second, we could not account for underlying changes in smoking and drinking. Changes in smoking and drinking patterns would have occurred even without the pandemic [45]. It would have been possible to account for ongoing changes if data on the previous year or individuals who were not affected by the pandemic were available, but the data used here were obtained from the first year of the Koreans’ Happiness Survey, and the entire South Korean population is affected by the pandemic. Third, the data were obtained from a self-report survey and are subject to measurement error. However, the impact of measurement error for the dependent variable on the main analyses is expected to be minimal since the dependent variable was operationalized as a three-category variable (“increased”, “decreased”, “no change”) rather than as a continuous variable reflecting a change in quantity. Moreover, the degree of measurement error is not likely to be associated with the dependent and independent variables (i.e., recall bias is not likely). Fourth, our outcome variable for the change in alcohol consumption combined the information on the change in per-week frequency and the change in per-occasion amount of consumption. These two patterns of consumption change are likely to indicate different phenomena, and this could not be reliably investigated. Fifth, while changes in smoking and drinking include information from the start of the pandemic to the time of the survey, the independent variables were recorded at the survey date. To identify associations between subgroups and behavioral changes, information on the independent variables immediately before the pandemic would have been more appropriate considering the temporality of the associations. While we could not entirely resolve this issue, we intentionally included independent variables that are relatively unlikely to change in the short term. Last, our findings are limited to alcohol drinking and smoking and do not reflect the overarching effect of the pandemic on overall health-harming behavior dynamics, especially other addictive behaviors or coping strategies. Further research should explore how behavioral changes in alcohol consumption and smoking occur alongside the changes in alternative health behaviors.

## 5. Conclusions

Our study provides evidence of changes in smoking and alcohol consumption during the COVID-19 pandemic in South Korea. We found that on average, the amount of smoking and drinking decreased during the pandemic. Notably, we also found that the differential increases in smoking and alcohol consumption occur among certain subgroups that may be more socioeconomically vulnerable to the impact of COVID-19: male gender, receipt of the basic living allowance, self-employment, unemployment, and chronic disease status in smoking and male gender, self-employment, living alone, having more offline friends, and having a chronic disease in drinking. Thus, social and economic policies to relieve their shock during the COVID-19 and to facilitate return to their normal life may be needed as the COVID-19 pandemic has been prolonged for years.

## Figures and Tables

**Table 1:** Characteristics of the nationally representative sample in South Korea.

Variable	Subheading	Overall	Smokers	Drinkers
Total number		13,824	2285	7912
Age (year)		45.88 ± 16.70	45.85 ± 14.93	44.94 ± 15.47
Sex (%)	Male	6903.5 (49.9)	2112.7 (92.5)	5064.1 (64.0)
Household income (%) (10,000 South Korean won)	0	196.4 (1.4)	8.2 (0.4)	78.8 (1.0)
	0–100	348.1 (2.5)	26.2 (1.1)	112.7 (1.4)
	100–200	822.8 (6.0)	104.5 (4.6)	412.4 (5.2)
	200–300	1865.4 (13.5)	269.1 (11.8)	937.0 (11.8)
	300–400	2804.5 (20.3)	458.5 (20.1)	1661.8 (21.0)
	400–500	2458.1 (17.8)	423.5 (18.5)	1459.3 (18.4)
	500–600	2009.2 (14.5)	348.9 (15.3)	1331.6 (16.8)
	600–700	1086.0 (7.9)	197.6 (8.6)	669.2 (8.5)
	700–800	585.5 (4.2)	57.6 (2.5)	341.3 (4.3)
	800–900	225.6 (1.6)	29.0 (1.3)	140.4 (1.8)
	900–1000	133.2 (1.0)	25.6 (1.1)	87.4 (1.1)
	1000 or more	1289.2 (9.3)	336.3 (14.7)	680.7 (8.6)
Basic living allowance (%)	Yes	173.8 (1.3)	21.5 (0.9)	57.9 (0.7)
Homeownership (%)	Homeowner	10,108.3 (73.1)	1572.7 (68.8)	5550.5 (70.1)
	Long-term rent with deposit	2912.2 (21.1)	527.4 (23.1)	1839.5 (23.2)
	Monthly rent	803.6 (5.8)	184.8 (8.1)	522.5 (6.6)
Employment status (%)	Regular employees	5926.2 (42.9)	1317.6 (57.7)	4110.8 (52.0)
	Self-employed	1894.7 (13.7)	466.5 (20.4)	1230.5 (15.6)
	Temporary workers	976.5 (7.1)	176.3 (7.7)	555.2 (7.0)
	Unemployed	5026.7 (36.4)	324.5 (14.2)	2016.0 (25.5)
Household type (%)	Living alone	981.4 (7.1)	207.5 (9.1)	548.3 (6.9)
Offline friends		4.00 (2.00–8.00)	5.00 (3.00–7.00)	5.00 (3.00–7.00)
Chronic disease status (%)	6 months or less	490.4 (3.5)	99.9 (4.4)	265.7 (3.4)
	More than 6 months	1266.4 (9.2)	222.4 (9.7)	610.4 (7.7)
SARS-CoV-2 positive (%)	Yes	14.9 (0.1)	0 (0.0)	9.2 (0.1)
	No	371.5 (2.7)	92.4 (4.0)	241.9 (3.1)
Smoking (%)	Smoker	2284.9 (16.5)	-	-
Alcohol consumption (%)	Drinker	7912.5 (57.2)	-	-
Change in smoking/drinking (%)	No change	-	1692.4 (74.1)	5370.1 (67.9)
	Increased	-	157.4 (6.9)	320.4 (4.0)
	Decreased	-	435.1 (19.0)	2221.9 (28.1)

The estimates are weighted by survey weights. Values are presented as number only, mean ± standard deviation, number (%), or median (interquartile range).

**Table 2:** Multinomial logistic regression results for the associations between various factors and changes in smoking and alcohol consumption.

Variable	Smoking		Alcohol Consumption	
	Increased	Decreased	Increased	Decreased
Male (ref: female)	3.20 [1.24, 8.26] *	0.97 [0.63, 1.48]	1.87 [1.41, 2.48] *	1.57 [1.39, 1.77] *
Basic living allowance (ref: no)	4.38 [1.36, 14.05] *	0.91 [0.19, 4.36]	1.01 [0.34, 2.99]	0.90 [0.49, 1.66]
Homeownership (ref: homeowner)				
Long-term rent with a deposit	1.19 [0.73, 1.93]	0.85 [0.61, 1.19]	1.09 [0.80, 1.49]	0.77 [0.66, 0.88] *
Monthly rent	0.95 [0.40, 2.27]	0.94 [0.54, 1.63]	1.27 [0.75, 2.15]	1.02 [0.80, 1.29]
Employment status (ref: regular employees)				
Self-employed	2.69 [1.73, 4.19] *	0.80 [0.55, 1.15]	2.20 [1.60, 3.03] *	1.03 [0.88, 1.21]
Temporary worker	1.24 [0.53, 2.88]	1.80 [1.11, 2.90] *	1.29 [0.74, 2.26]	1.28 [1.03, 1.60] *
Unemployed	3.26 [1.65, 6.44] *	1.53 [0.98, 2.40]	1.34 [0.92, 1.96]	0.89 [0.76, 1.04]
Living alone (ref: living with someone)	1.34 [0.67, 2.66]	0.56 [0.32, 0.98] *	2.16 [1.40, 3.33] *	0.94 [0.74, 1.19]
Offline friends	1.04 [0.99, 1.10]	0.95 [0.91, 0.99] *	1.04 [1.01, 1.08] *	1.00 [0.99, 1.02]
Chronic disease (ref: none)				
6 months or less	2.17 [1.11, 4.23] *	1.43 [0.79, 2.57]	2.16 [1.29, 3.61] *	1.63 [1.23, 2.16] *
More than 6 months	2.32 [1.37, 3.95] *	0.62 [0.37, 1.02]	2.26 [1.51, 3.36] *	1.04 [0.83, 1.29]
Total number	2179		7662	

* Statistically significant (*p* < 0.05). Values are presented as odds ratio (95% confidence interval) or number only. Age and household income were included as spline functions, and their estimated parameters are visualized for interpretation (Figure 2 and Figure 3). Coefficients for intercept, age, household income, and location of residence are omitted.

## Data Availability

The data sets used and analyzed for this study are available from the corresponding authors on reasonable request.

## Supplementary Materials

The following are available online at https://www.mdpi.com/article/10.3390/ijerph19052802/s1, Table S1: Multinomial logistic regression results for the associations between a range of factors and the changes in smoking and alcohol consumption using the categorical household income variable, Table S2: Multinomial logistic regression results for the associations between a range of factors and the changes in smoking and alcohol consumption among those aged 20 or older.
