# Supplementary material for: Employment, Economic, and Sociodemographic Factors Associated with Changes in Smoking and Drinking Behaviors during the COVID-19 Pandemic in South Korea
Source: Int J Environ Res Public Health. 2022 Feb 28;19(5):2802. doi: 10.3390/ijerph19052802 (PMC8910664; doi:10.3390/ijerph19052802)
Supplement: Supplementary file 1 [file ijerph-19-02802-s001.zip › ijerph-1574459-supplementary.pdf]

Supplementary Table S1 Multinomial logistic regression results for the associations between a range of factors and the changes in smoking and alcohol consumption using the categorical household income variable

	Smoking		Alcohol consumption	
	Increased	Decreased	Increased	Decreased
Male (ref: female)	**3.19 [1.24; 8.24]**	0.98 [0.64; 1.51]	**1.89 [1.42; 2.50]**	**1.58 [1.40; 1.78]**
Household income (ref: more than 10 million won)				
9-10 million won	0.98 [0.19; 5.14]	0.47 [0.16; 1.38]	1.09 [0.24; 5.03]	1.19 [0.72; 1.97]
8-9 million won	0.46 [0.09; 2.35]	**0.22 [0.06; 0.81]**	1.62 [0.63; 4.17]	0.71 [0.47; 1.08]
7-8 million won	0.47 [0.14; 1.63]	**0.30 [0.14; 0.64]**	1.85 [0.89; 3.84]	**0.73 [0.53; 1.00]**
6-7 million won	0.58 [0.25; 1.37]	**0.31 [0.18; 0.53]**	0.92 [0.46; 1.84]	**0.48 [0.36; 0.62]**
5-6 million won	0.49 [0.23; 1.05]	**0.20 [0.12; 0.32]**	1.14 [0.62; 2.11]	**0.63 [0.50; 0.79]**
4-5 million won	0.54 [0.26; 1.12]	**0.24 [0.15; 0.37]**	1.24 [0.68; 2.24]	**0.53 [0.42; 0.66]**
3-4 million won	0.52 [0.25; 1.10]	**0.25 [0.16; 0.39]**	0.88 [0.48; 1.61]	**0.46 [0.36; 0.57]**
2-3 million won	0.72 [0.33; 1.56]	**0.32 [0.19; 0.54]**	1.31 [0.71; 2.43]	**0.63 [0.49; 0.80]**
1-2 million won	0.39 [0.13; 1.21]	**0.18 [0.08; 0.41]**	0.93 [0.42; 2.05]	**0.64 [0.46; 0.88]**
less than 1 million won	1.27 [0.25; 6.53]	0.45 [0.12; 1.66]	0.61 [0.15; 2.41]	1.08 [0.66; 1.77]
no income	1.13 [0.09; 14.12]	0.41 [0.07; 2.46]	1.70 [0.44; 6.53]	1.05 [0.60; 1.82]
Basic living allowance (ref: no)	**4.32 [1.34; 13.96]**	0.93 [0.19; 4.49]	1.08 [0.36; 3.21]	0.89 [0.48; 1.64]
Homeownership (ref: homeowner)				
Long-term rent with deposit	1.21 [0.74; 1.97]	0.86 [0.62; 1.21]	1.07 [0.78; 1.46]	**0.77 [0.66; 0.88]**
Monthly rent	0.95 [0.39; 2.28]	0.92 [0.53; 1.59]	1.28 [0.75; 2.16]	1.01 [0.79; 1.28]
Employment status (ref: regular employees)				
Self-employed	**2.72 [1.74; 4.25]**	0.81 [0.56; 1.17]	**2.22 [1.61; 3.06]**	1.04 [0.88; 1.21]
Temporary worker	1.31 [0.56; 3.06]	**1.85 [1.14; 3.00]**	1.32 [0.75; 2.31]	**1.28 [1.03; 1.60]**
Unemployed	**3.39 [1.70; 6.76]**	**1.59 [1.01; 2.51]**	1.39 [0.95; 2.02]	0.90 [0.76; 1.05]
Living alone (ref: living with someone)	1.34 [0.66; 2.70]	**0.57 [0.32; 0.99]**	**2.20 [1.42; 3.41]**	0.92 [0.72; 1.17]
Offline friends	1.04 [0.99; 1.10]	**0.95 [0.91; 0.99]**	**1.04 [1.00; 1.07]**	1.00 [0.99; 1.02]
Chronic disease (ref: none)				
6 months or less	**2.21 [1.13; 4.33]**	1.42 [0.78; 2.57]	**2.19 [1.31; 3.67]**	**1.64 [1.23; 2.18]**
More than 6 months	**2.38 [1.40; 4.06]**	0.65 [0.39; 1.08]	**2.26 [1.52; 3.38]**	1.03 [0.83; 1.28]
N	2179		7662	

Estimates with $p < 0.05$ are bolded.
Age and household income were included as spline functions. Coefficients for intercept, age, household, and location of residence are omitted.

Supplementary Table S2 Multinomial logistic regression results for the associations between a range of factors and the changes in smoking and alcohol consumption among those aged 20 or older

	Smoking		Alcohol consumption	
	Increased	Decreased	Increased	Decreased
Male (ref: female)	**3.22 [1.25; 8.31]**	0.95 [0.62; 1.46]	**1.88 [1.41; 2.49]**	**1.60 [1.42; 1.81]**
Basic living allowance (ref: no)	**4.38 [1.36; 14.07]**	0.90 [0.19; 4.32]	1.01 [0.34; 3.01]	0.90 [0.19; 4.32]
Homeownership (ref: homeowner)				
Long-term rent with deposit	1.19 [0.73; 1.94]	0.85 [0.61; 1.19]	1.09 [0.79; 1.49]	**0.77 [0.67; 0.89]**
Monthly rent	0.94 [0.39; 2.25]	0.84 [0.47; 1.48]	1.29 [0.76; 2.18]	1.01 [0.79; 1.28]
Employment status (ref: regular employees)				
Self-employed	**2.70 [1.73; 4.20]**	0.79 [0.55; 1.14]	**2.16 [1.57; 2.97]**	1.03 [0.88; 1.21]
Temporary worker	1.24 [0.53; 2.89]	**1.79 [1.11; 2.89]**	1.28 [0.73; 2.25]	**1.26 [1.01; 1.57]**
Unemployed	**3.30 [1.68; 6.50]**	1.55 [0.99; 2.44]	1.35 [0.92; 1.97]	0.91 [0.77; 1.06]
Living alone (ref: living with someone)	1.34 [0.67; 2.66]	0.59 [0.34; 1.02]	**2.14 [1.38; 3.30]**	0.93 [0.73; 1.18]
Offline friends	1.04 [0.99; 1.10]	**0.95 [0.91; 0.99]**	**1.04 [1.00; 1.07]**	1.00 [0.99; 1.02]
Chronic disease (ref: none)				
6 months or less	**2.16 [1.11; 4.22]**	1.43 [0.79; 2.58]	**2.18 [1.31; 3.65]**	**1.63 [1.23; 2.17]**
More than 6 months	**2.32 [1.36; 3.94]**	0.61 [0.37; 1.02]	**2.25 [1.51; 3.36]**	1.03 [0.83; 1.28]
N	2168		7550	

Estimates with $p < 0.05$ are bolded.
Age and household income were included as spline functions. Coefficients for intercept, age, household, and location of residence are omitted.
